# Thyroid and cortisol endocrinopathies and survival in cancer patients treated with immune checkpoint inhibitors in UAE

**DOI:** 10.1210/jendso/bvag037

**Published:** 2026-02-17

**Authors:** Mohamed Alqedra, Bara Fahmayee, Lina Wahba, Jawaher Ansari, Romona D Govender, Saif Al-Shamsi, Raya Almazrouei

**Affiliations:** Department of Internal Medicine, Tawam Hospital, Al Ain, United Arab Emirates; Department of Internal Medicine, Tawam Hospital, Al Ain, United Arab Emirates; Department of Pharmacy, Tawam Hospital, Al Ain, United Arab Emirates; Department of Medical Oncology, Tawam Hospital, Al Ain, United Arab Emirates; Department of Family Medicine, College of Medicine and Health Sciences, United Arab Emirates University, United Arab Emirates; Department of Internal Medicine, College of Medicine and Health Sciences, United Arab Emirates University, United Arab Emirates; Department of Internal Medicine, College of Medicine and Health Sciences, United Arab Emirates University, United Arab Emirates; Division of Endocrinology, Tawam Hospital, Al Ain, United Arab Emirates

**Keywords:** immune checkpoint, endocrinopathy, cortisol, thyroid, adrenal insufficiency, pituitary

## Abstract

**Background:**

Immune checkpoint inhibitors (ICIs) are associated with immune-related adverse events (irAEs) of which endocrinopathies are among the most frequent. This study aimed to identify thyroid and cortisol-related endocrine-related adverse events (ERAEs) in a cohort of patients treated with ICIs and to examine survival differences between patients who developed endocrinopathies and those who did not.

**Methods:**

We conducted a retrospective review of electronic medical records of adult patients who received ICIs between 2018 and 2023. Data were collected specifically on thyroid and cortisol-related ERAEs.

**Results:**

Among 616 patients, 59 (9.6%) developed thyroid or cortisol-related ERAEs. The mean time to onset was 22.7 weeks. All thyroid-related events were post-thyroiditis hypothyroidism (*n* = 55), while all cortisol-related events were due to adrenocorticotropic hormone (ACTH) deficiency (*n* = 11). The majority of these events occurred in patients treated with anti–PD-1 agents, the most commonly used therapy in this cohort. Patients who developed ERAEs demonstrated improved overall survival during the follow-up period compared to those without endocrine toxicity.

**Conclusion:**

In this cohort, systematic monitoring identified thyroid- and cortisol-related ERAEs in 9.6% of patients, consisting exclusively of post-thyroiditis hypothyroidism and ACTH deficiency. The occurrence of these endocrinopathies was associated with a favorable survival trend, underscoring the importance of early recognition and management of endocrine irAEs in patients receiving ICIs.

Immune checkpoint inhibitors (ICIs) have transformed cancer therapy, demonstrating robust response rates and improved survival across several malignancies, including melanoma, renal cell carcinoma, and lung cancer. ICIs are classified into 3 major categories: cytotoxic T-lymphocyte-associated protein 4 (CTLA-4) inhibitors, programmed cell death protein-1 (PD-1) inhibitors, and programmed death ligand-1 (PD-L1) inhibitors [1]. These agents act by enhancing T-lymphocyte–mediated cytotoxicity and augmenting the adaptive immune response. However, this immune activation also predisposes patients to organ-specific autoimmune toxicities, including endocrine-related adverse events (ERAEs).

Among the most common ERAEs are thyroid dysfunction and anterior pituitary disorders, particularly hypophysitis leading to glucocorticoid deficiency [2]. Less frequently reported endocrine complications include type 1 diabetes mellitus, primary adrenal insufficiency, and hypoparathyroidism [3]. The incidence of ERAEs varies by the type of ICI used, with rates ranging from 12% to 40% [4]. These events typically occur within the first few months of therapy, although delayed onset during or after treatment has also been described [5]. Notably, the development of irAEs has been associated with improved progression-free survival, overall survival, and treatment response [4].

In this study, we report on thyroid and cortisol-related ERAEs among patients treated with ICIs at a tertiary care center in the United Arab Emirates (UAE). We focused specifically on these 2 axes due to robust routine monitoring in our oncology practice. We also evaluated survival outcomes in patients who developed these ERAEs compared to those who did not.

## Methods

This retrospective study was conducted at Tawam Hospital, Al Ain, UAE. We identified all patients aged ≥16 years who were prescribed ICIs between 1 January 2018 and 31 March 2023 through oncology pharmacy records. Included ICIs were atezolizumab, durvalumab, nivolumab, pembrolizumab, and the ipilimumab-nivolumab combination. We reviewed medical records to extract demographic and clinical data. The data lock date was 31 July 2024.

Patients were excluded if ICI therapy was not initiated or if they died prior to treatment. All included patients had thyroid function and cortisol levels measured in the morning prior to ICI initiation and before each treatment cycle routinely. Other endocrine tests, such as full pituitary panel or HbA1c, were infrequently performed and thus not analyzed. Consequently, this study reports only on thyroid- and cortisol-related ERAEs. Additionally, pituitary imaging is not performed in our patients and therefore not reported here.

All patients who developed thyroid- or cortisol-related adverse events during immune checkpoint inhibitor therapy were carefully evaluated by certified endocrinologists. Diagnostic work-up followed internationally accepted standards and incorporated biochemical cut-offs, repeat confirmatory measurements, and, when indicated, dynamic assessment with the short Synacthen (cosyntropin) stimulation test [3].

Thyroid ERAEs were defined as new-onset dysfunction after ICI treatment, including central hypothyroidism, primary hypothyroidism (further specified as post-thyroiditis hypothyroidism where applicable), subclinical thyroid dysfunction, and hyperthyroidism. Central hypothyroidism was defined as low free T4 with low or inappropriately normal TSH. Primary hypothyroidism was defined by elevated TSH and low free T4, with review of cases to determine whether hypothyroidism occurred following thyroiditis. Subclinical hypothyroidism was defined as TSH >10 mU/L with normal free T4. Thyroid antibodies were not routinely measured [3].

Adrenal ERAEs were classified as either primary adrenal insufficiency (low cortisol with high adrenocorticotropic hormone [ACTH]) or secondary (central) adrenal insufficiency (low cortisol with low or inappropriately normal ACTH) in the absence of prior steroid exposure. In our cohort, all cases were consistent with secondary adrenal insufficiency. Paired early morning cortisol and ACTH testing was the main diagnostic approach; affected patients showed cortisol concentrations that were low (often <100 nmol/L) with ACTH levels that were inappropriately low or within the lower reference range. When morning cortisol results were indeterminate, repeat testing or Synacthen stimulation was performed to confirm the diagnosis [3]. This structured and standardized approach ensured robust diagnostic accuracy and minimized misclassification of endocrine-related adverse events. All hormone levels were measured using Roche-Cobas Generation II assays. Cancer types were categorized by anatomical site. This study was approved by the Tawam Human Research Ethics Committee (MF20258-2023-937), which waived the requirement for informed consent due to its retrospective nature and anonymized data.

### Statistical analysis

Continuous variables are presented as mean ± SD or median with interquartile range; categorical variables as frequencies and percentages. Fisher's exact test was used for categorical comparisons. A Cox proportional hazards model evaluated predictors of ERAEs, including age, sex, and ICI type. Kaplan–Meier survival analysis estimated survival probabilities, and differences between groups were assessed using the log-rank test. Analyses were performed in R version 4.4.1 (The R Foundation, Vienna, Austria). A *P*-value < .05 was considered statistically significant.

## Results

We identified 616 patients who received ICIs. Baseline characteristics are presented in Table 1. The mean age was 56.2 ± 14 years, and 57.8% were male. Patients were primarily from the Middle East (54.4%), followed by Asia (23.7%) and the UAE (15.7%). Pre-existing thyroid disease was present in 26 patients (4.2%), predominantly hypothyroidism (3.9%). Diabetes mellitus was present in 15.9%, including one patient with type 1 diabetes. Lung cancer was the most common malignancy (27.6%).

**Table 1:** Baseline characteristics, *n* = 616

Variable	*N* (%)
Age (SD)	56.2 (14.0)
Gender	
Female	260 (42.2)
Male	356 (57.8)
Ethnicity	
African	29 (4.7)
Asian	146 (23.7)
Middle East	335 (54.4)
UAE	97 (15.7)
Others	9 (1.5)
Baseline endocrine disease	
Nil	492 (79.9)
Graves' disease	2(0.3)
Hypothyroidism	22 (3.6)
DM	98 (15.9)
Hypothyroidism and DM	2 (0.3)
Malignancy type or site	
Breast cancer	64 (10.4)
Genitourinary	97 (15.7)
Gastrointestinal tract	66 (10.7)
Gynecological	69 (11.2)
Head and neck	79 (12.8)
Hepatobiliary	21 (3.4)
Lung	170 (27.6)
Lymphoma	11 (1.8)
Melanoma	26 (4.2)
Others	13 (2.1)
Immune checkpoint inhibitor used	
Anti CTLA-4	19 (3.1)
Anti PD-1	478 (77.6)
Anti PD-1 + anti CTLA-4	8 (1.3)
Anti PD-L1	111 (18.0)

Abbreviations: CTLA-4, cytotoxic T-lymphocyte-associated protein 4; N; number; PD-1, programmed cell death protein-1; PD-L1, programmed death ligand-1; SD, standard deviation.

The most frequently administered ICI was anti-PD-1 (77.6%), followed by anti-PD-L1 (18%). As shown in Table 2, thyroid- and cortisol-related ERAEs were diagnosed in 59 patients (9.6%). Post-thyroiditis hypothyroidism developed in 55/59 patients; no patients developed hyperthyroidism. ACTH deficiency (central adrenal insufficiency) was observed in 11 patients, of whom 7 also had post-thyroiditis hypothyroidism. Most ERAEs occurred in patients receiving anti-PD-1 therapy. Mean time to ERAE onset was 22.7 ± 15.3 weeks overall, 22.3 ± 15.0 weeks for thyroiditis-related hypothyroidism, and 34.0 ± 18.3 weeks for ACTH deficiency. In multivariable analysis (Table 3), use of anti-PD-L1 was the only significant predictor of ERAE development.

**Table 2:** Frequency of endocrine-related adverse events (ERAE)

Any ERAE, *n* (%) *n* = 615	
No	556 (90.4)
Yes	59 (9.6)
ERAE time onset (weeks) *n* = 59	
Mean (SD)	22.7 (15.3)
Median (IQR)	20.0 (22.0)
Post thyroiditis hypothyroidism, *n* (%) *n* = 55	
Anti CTLA-4	4 (7.3)
Anti PD-1	46 (83.6)
Anti PD-1 + anti CTLA-4	1 (1.8)
Anti PD-L1	4 (7.3)
ACTH deficiency, *n* (%) *n* = 11	
Anti CTLA-4	0 (0.0)
Anti PD-1	8 (72.7)
Anti PD-1 + anti CTLA-4	2 (18.2)
Anti PD-L1	1 (9.1)
TFT at the time of hypothyroid phase diagnosis, median (IQR) *n* = 51	
TSH (mIU/L)	56 (18.6-138)
Free T4 (pmol/L)	5.4 (1.2-10)
ACTH and Cortisol levels at the time of diagnosis, median (IQR) *n* = 11	
ACTH (pmole/L)	0.3 (0.3-1.1)
Cortisol (nmol/L)	15 (3-75)

Abbreviations: ACTH, adrenocorticotropic hormone; CTLA-4, cytotoxic T-lymphocyte-associated protein 4; ERAE, endocrine-related adverse events; FT4, free thyroxine; IQR, interquartile range; PD-1, programmed cell death protein-1; PD-L1, programmed death ligand-1; TFT, thyroid function tests; TSH, thyroid-stimulating hormone.

Laboratory reference ranges: TSH 0.35-4.6 mIU/L; free T4 9-19 pmol/L; ACTH 1.6-13.9 pmol/L; cortisol 64-536 nmol/L.

**Table 3:** Predictors of endocrine-related adverse events (ERAE), *n* = 615

Variables	HR	95% CI	*P*-value
Age at starting of ICPs	0.99	0.97 to 1.01	.157
Gender (male)	0.98	0.58 to 1.65	.927
Immune checkpoint inhibitor used			
Anti CTLA-4	Reference		
Anti PD-1	0.62	0.22 to 1.73	.359
Anti PD-1 + anti CTLA-4	1.48	0.27 to 8.24	.655
Anti PD-L1	0.23	0.06 to 0.96	.043

Abbreviations: CTLA-4, cytotoxic T-lymphocyte-associated protein 4; ERAE, endocrine-related adverse events; HR, hazard ratio; ICP, immune checkpoint inhibitor; PD-1, programmed cell death protein-1; PD-L1, programmed death ligand-1.

**Figure 1:**
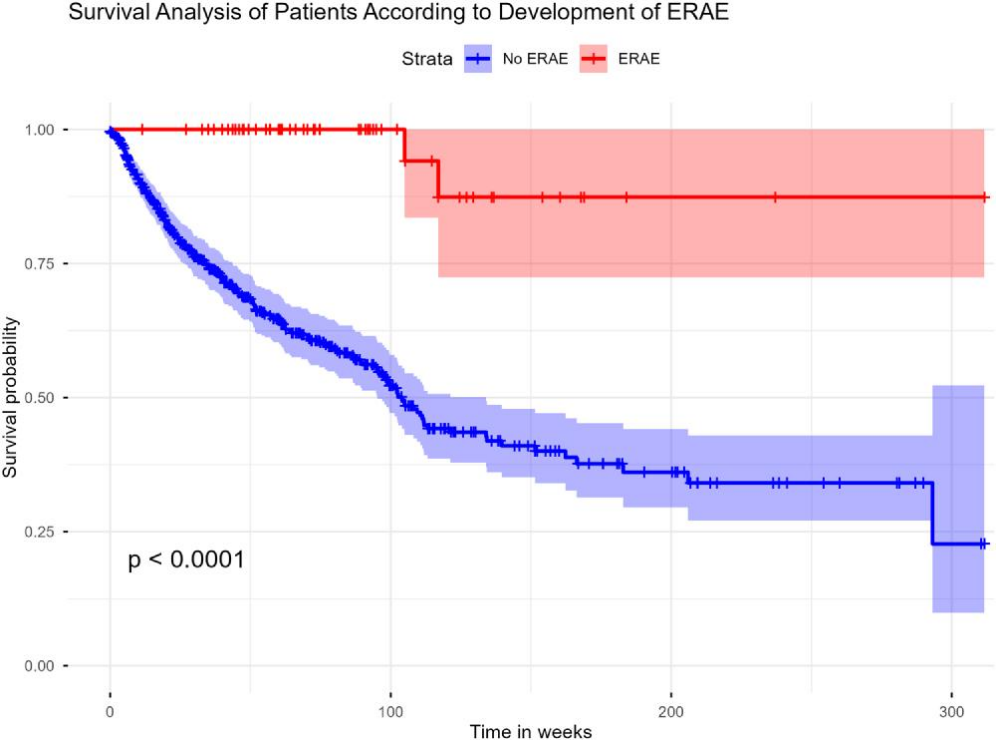
Kaplan–Meier survival analysis of patients treated with immune checkpoint inhibitors, stratified by the development of endocrine-related adverse events (ERAEs).

Kaplan–Meier survival analysis (Fig. 1) showed significantly better survival among patients with ERAEs compared to those without (log-rank *P* < .0001). Survival probabilities remained higher over a 312-week follow-up in the ERAE group, whereas the non-ERAE group showed a steeper decline.

## Discussion

This single-center study from the UAE evaluated both thyroid and cortisol-related ERAEs among cancer patients undergoing treatment with ICIs and also explored their association with survival outcomes. The emergence of immune-related adverse events (irAEs), such as thyroid and cortisol-related ERAEs, is not only an anticipated yet challenging aspect of their clinical use, but is also recognized as a potential marker of improved survival outcomes. Patients in this study, who experienced ERAEs, demonstrated a statistically significant higher survival probability compared to those without ERAEs. While similar patterns have been reported globally [6-8], our study provides region-specific insight from the UAE, where such data remain scarce. This contributes valuable context to the Middle Eastern and Gulf literature, where varying rates of ERAEs and survival outcomes suggest regional differences in monitoring practices, treatment regimens, and population characteristics [9-11].

The overall ERAE incidence of 9.6% in this study is comparable to rates reported in large Western datasets and meta-analyses, where endocrine toxicities typically range around 9-13% [4, 12-14], which is notably lower than the rates documented in neighboring Gulf countries: Oman 28% [9], Saudi Arabia 20.85% [10], and Qatar 26.7% [11]. Several factors may explain these differences [9-11]. First, some regional studies included a broader spectrum of endocrine toxicities, such as pituitary dysfunction, diabetes, and gonadal abnormalities whereas our analysis focused specifically on thyroid and cortisol adverse outcomes. Second, the inclusion criteria, as well as differences in screening intensity and diagnostic methods varied; for example, assessment for pituitary hormones or MRI evaluation by some of the centers influenced their results. Third, most of the other studies lack standardized testing protocols for screening potential endocrine adverse effects. Forth, differences in ethnicity, baseline comorbidities, and therapeutic regimens (combination checkpoint blockade vs monotherapy) may all contribute to variability.

Thyroid dysfunction was the most frequent endocrine toxicity in our results, consistent with international and regional data [9, 10, 15]. The majority of cases occurred in patients receiving anti–PD-1 inhibitors, which constituted 77.6% of our treatments. Higher PD-L1 expression in thyroid follicular cells may predispose patients to immune-mediated thyroiditis when treated with these agents [16]. In contrast, ACTH deficiency, observed in 1.79% of our patients, was rare but clinically significant. This lower rate relative to reports among Japanese patients [17] and other cohorts [10] may reflect differences in diagnostic thresholds, imaging frequency, or management protocols. Although pituitary MRI was not routinely performed among our patients, current evidence suggests limited benefit and therefore may not be necessary in most cases of immune checkpoint inhibitor–related hypophysitis or ACTH deficiency [18].

Mean onset of ERAEs in our cohort was approximately 22.7 weeks, similar to previous reports [9]. Prior reports describing ERAEs, documented their development over a wide time frame, averaging between 1 and 2 months [19, 20] and with some events occurring even later [21] highlighting the importance of vigilant hormonal monitoring particularly during the later phases of ICI therapy. The inclusion of cortisol, ACTH, TSH, and free T4 levels at the time of diagnosis strengthens the clinical characterization of these events, facilitating differentiation between primary and secondary etiologies and thus supporting tailored monitoring and management.

Identifying predictive biomarkers for ERAEs remains an ongoing challenge. Although anti–PD-L1 therapy emerged as an independent predictor in our cohort, no biomarker has shown consistent reliability across studies [22]. Future research should integrate longitudinal hormonal assessments, immunologic profiling, and pharmacogenetic data to clarify susceptibility patterns and guide personalized monitoring.

### Strengths and limitations

This study's strengths include its extended follow-up period and the implementation of a rigorous protocol for monitoring thyroid hormones and cortisol levels, enabling precise characterization of adverse event patterns. The diverse, real-world patient population enhances the applicability of our findings to similar clinical settings. As retrospective study, it is limited by lack of data on other endocrinopathies due to lack of robust testing and insurance coverage. Additionally, data on non-endocrine adverse events were not collected. Despite these limitations, our results highlight the critical importance of standardized surveillance protocols for early detection and management of endocrine-related adverse events. Educating patients about potential endocrine toxicities—and their potential association with positive treatment outcomes—may improve adherence and support shared decision-making.

## Conclusion

In summary, thyroid and cortisol-related ERAEs occurred in 9.6% of cancer patients receiving ICIs in this UAE cohort and were associated with improved survival outcomes. Although the incidence was lower than in neighboring Gulf countries, the findings reinforce the importance of vigilant endocrine monitoring, region-specific reporting, and awareness that these immune-related endocrinopathies may serve not only as adverse effects but also as potential prognostic indicators of treatment efficacy.

## Data Availability

Restrictions apply to the availability of some or all data generated or analyzed during this study to preserve patient confidentiality or because they were used under license. The corresponding author will on request detail the restrictions and any conditions under which access to some data may be provided.

## Abbreviations

ACTH: adrenocorticotropic hormone
CTLA-4: cytotoxic T-lymphocyte-associated protein 4
ERAE: endocrine-related adverse events
FT4: free thyroxine
ICI: immune checkpoint inhibitor
ICP: immune checkpoint inhibitor
HR: hazard ratio
IQR: interquartile range
irAE: immune-related adverse event
PD-1: programmed cell death protein-1
PD-L1: programmed death ligand-1
SD: standard deviation
TFT: thyroid function tests
TSH: thyroid-stimulating hormone
